# CASE REPORT Bilateral Paradoxically Symptomatic Luno-triquetral Coalition: A Case Report

**Published:** 2010-06-23

**Authors:** Oliver Lotter, Stephane Stahl, Oliver Luz, Matthias Pfau, Hans-Eberhard Schaller

**Affiliations:** Department of Plastic, Hand and Reconstructive Surgery, Burn Center, BG Trauma Center, Eberhard-Karls, University Tübingen, Germany

## Abstract

**Objective:** While bony luno-triquetral coalitions are known to be asymptomatic, fibro-cartilage unions can cause ulnar-sided wrist pain. The purpose of this case report is to present a paradox clinical constellation of bilateral luno-triquetral coalition. Furthermore, recommendations for proper diagnosis and treatment options will be discussed. **Methods:** The case of a 21-year-old female patient is reported, where a bony coalition of one side caused wrist pain and the contralateral fibro-cartilage bonding was asymptomatic. **Results:** Because of the stable bony coalition in the symptomatic wrist, we refused to undertake a luno-triquetral fusion and continued conservative treatment with the option of wrist denervation. **Conclusions:** Consequently, not only incomplete but also complete luno-triquetral coalitions can cause wrist pain. Unfortunately, no clear biomechanical explanation is available for this finding.

Luno-triquetral coalition is a congenital carpal anomaly that is most often diagnosed as an incidental finding in asymptomatic patients and can be associated with other synostoses or malformations. It is the most common coalition representing nearly 90% of all carpal fusions, followed by the capito-hamate coalition with 5.6% of all carpal fusions.^1^ The prevalence in the general population averages 0.1% but is thought to be 100 times higher in Africans.^2^ This anomaly is found more often in females with a ratio of 2:1.^3^,^4^ Embryological carpal coalition represents a failure of cavitation of the cartilaginous hand bud precursor during the fourth to eighth week of gestation, which later develops to an osseous, fibrous, or cartilaginous union.^5^ It is classified according to the degree of union and is often found bilateral. According to literature, the biggest collective has been reported from Senegal, where 32 luno-triquetral coalitions in 20 patients were examined in a retrospective study. The complete form (type III of Minaar's classification) was the most frequent (46.8%), followed by the incomplete osseous fusion with a distal notch (28.1%).^6^,^7^ While synostosis of the lunate and the triquetrum is known to be asymptomatic, fibro-cartilaginous bonding can present an uncommon cause for ulnar-sided wrist pain.^8^,^9^ This is the case of a bilateral luno-triquetral coalition causing symptoms only on the side of a stable fusion, whereas the contra lateral wrist, with an incomplete fibro-cartilaginous component, is asymptomatic.

## CASE PRESENTATION

**Figure 1:**
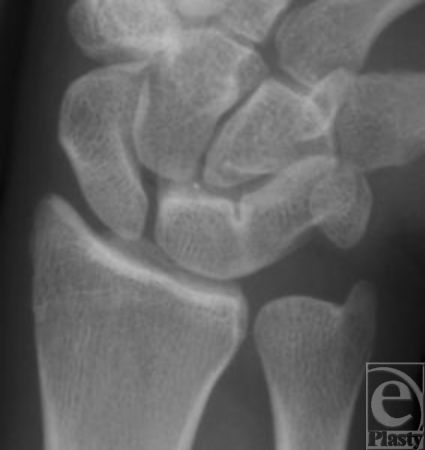
Anteroposterior radiograph of the right painful wrist showing a luno-triquetral coalition Minaar type II.

**Figure 2:**
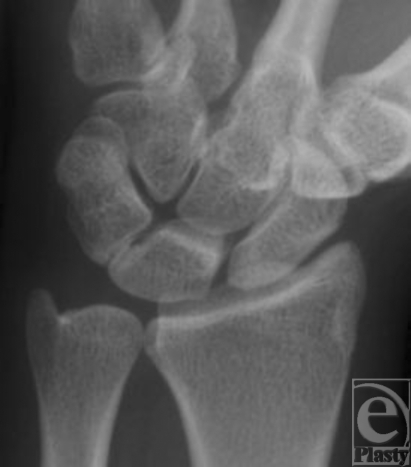
Anteroposterior radiograph of the left asymptomatic wrist showing a luno-triquetral coalition Minaar type I.

**Figure 3:**
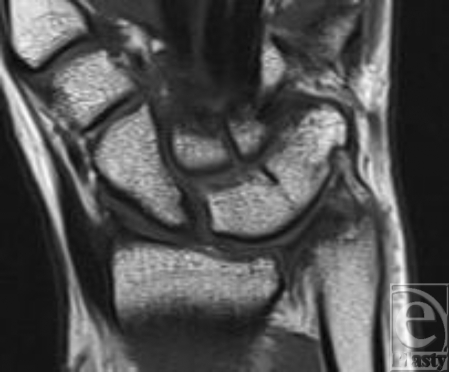
T1 weighted turbo-spin-echo coronal shows the luno-triquetral coalition Minaar type II confirming the stable proximal fusion of the bones with a distal notch of the right wrist.

**Figure 4:**
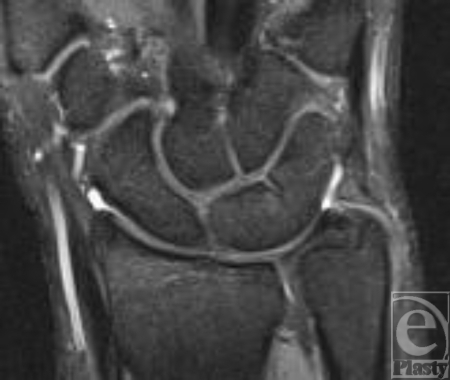
Proton-density-weighted technique in combination with fat saturation coronal shows no bone edema on the right side.

A 21-year-old, right-hand-dominant, white, female student presented in our policlinic with right-sided wrist pain, since 2 years, without any previous trauma. She described an ulno-dorsal intermittent stabbing pain on twisting her hand, particularly when bearing loads during sports. Physical examination revealed tenderness located on the luno-triquetral joint. No swelling or ache in other locations of the right hand could be found. The left wrist was completely asymptomatic on examination and there was no pathologic “shuck” and “shear” test between the lunate and the triquetrum.^10^ The range of motion of both wrists was normal. Plain radiographs showed a right-sided osseous fusion of the lunate and triquetrum with a distal notch according to Minaar's classification type II (Fig 1) and an incomplete proximal fibro-cartilaginous coalition type I on the left (Fig 2).^2^ Radial and ulnar clenched fist views of both wrists did not reveal any widening of the scapho-lunate joint space. Magnetic resonance imaging of the right wrist did not show any pathology, with the exception of a small volar ganglion, 5 mm in diameter, in the radio-scaphoid region as an ancillary finding (Figs 3 and 4). We recommended the patient to continue physiotherapy, because she rated the right-sided wrist pain to be of medium intensity (4/10 points on the Graphical Rating Scale) and kept the option of wrist denervation open for the future, in case of aggravation.

## DISCUSSION

The joint between lunate and triquetrum is the most common location for carpal coalition. Depending on the degree of cellular apoptosis, different types of bonding can develop, which can range anywhere between complete coalition and normal joint development. Minaar classified this anatomical variation in 4 types. Type I represents an incomplete fusion similar to a pseudarthrosis (fibro-cartilage coalition), type II an incomplete osseous fusion, type III a complete osseous fusion (os lunato-triquetrum), and type 4 a complete osseous fusion associated with other carpal anomalies.^7^,^11^

For proper diagnosis, we suggest to perform anteroposterior and lateral radiographs as well as radial and ulnar clenched fist views of both wrists. Scapho-lunate joint space widening is commonly associated with luno-triquetral coalition but was not present in our case.^12^ Cineradiography can be added if abnormal motion of the carpal bones in relation to each other is suspected. We recommend a computed tomographic scan in case of difficulty evaluating the stability of the bonding between the affected carpal bones in symptomatic wrists. In our opinion, the only reason to justify magnetic resonance imaging is to exclude concomitant pathologies of the wrists, which are to be evaluated before operative treatment of luno-triquetral coalitions.

Contrary to the common opinion that synostoses exclude motion between the affected bones and therefore are asymptomatic, our patient suffered from painful wrist movement on the side of the complete bony fusion.^8^,^13^ Paradoxically, the patient did not complain about left-sided wrist pain where we diagnosed the incomplete, pseudoarthrosis-like fibro-cartilage coalition, where one would expect the patient to primarily have wrist pain.

As no motion is expected between the lunate and the triquetrum in Minaar's classification type II-IV patients, luno-triquetral arthrodesis is not recommended in any case.^3^ However, deficient cartilage formation between incomplete separated carpal bones in Minaar's type I patients can result in symptoms analogous to those of degenerative arthritis.^13^ In this case, wrist denervation, fusion of the affected bones, or both should be considered. Till now, no clear biomechanical model exists to explain firm symptomatic luno-triquetral coalitions. It is well known, from other pathologies of the wrist, that the disturbance of the coordinated movement of the carpal bones may result in instability, which in most cases is hardly detectable by the diagnostic possibilities named earlier.^14^,^15^ Lunate and triquetrum show their maximal translation in ulnar deviation.^16^^-^18 In the presence of luno-triquetral synostosis, the relative movement is disturbed and the proximal row rotates as a unit resulting in instability.^19^

Paradoxically, carpal fusions can result in instability of the wrist, with an increased risk for arthritis, even though carpal fusion is accepted as a treatment option for carpal instability. Therefore, we would recommend one to exhaust conservative treatment, such as physiotherapy, and offer a wrist denervation in case of substantial, pain-related, reduced quality of life. Only in the presence of carpal arthritis is a partial arthrodesis or, if pronounced, total wrist arthrodesis—a treatment alternative to wrist denervation.
